# Computational methods and data resources for predicting tumor neoantigens

**DOI:** 10.1093/bib/bbaf302

**Published:** 2025-07-03

**Authors:** Xiaofei Zhao, Lei Wei, Xuegong Zhang

**Affiliations:** MOE Key Lab of Bioinformatics, Bioinformatics Division of BNRIST and Department of Automation, Tsinghua University, Beijing 100084, China; MOE Key Lab of Bioinformatics, Bioinformatics Division of BNRIST and Department of Automation, Tsinghua University, Beijing 100084, China; MOE Key Lab of Bioinformatics, Bioinformatics Division of BNRIST and Department of Automation, Tsinghua University, Beijing 100084, China; School of Life Sciences and School of Medicine, Tsinghua University, Beijing 100084, China

**Keywords:** neoantigen, neoepitope, cancer genomics, immunoinformatics, personalized immunotherapy

## Abstract

Neoantigens are tumor-specific antigens presented exclusively by cancer cells. These antigens are recognized as nonself by the host immune system, thereby eliciting an antitumor T-cell response. This response is significantly enhanced through neoantigen-based immunotherapies, such as personalized cancer vaccines. The repertoire of neoantigens is unique to each cancer patient, necessitating neoantigen prediction for designing patient-specific immunotherapies. This review presents the computational methods and data resources used for neoantigen prediction, as well as the prediction-associated challenges. Neoantigen prediction typically uses human leukocyte antigen typing, RNA-seq transcript quantification, somatic variant calling, peptide–major histocompatibility complex (pMHC) presentation prediction, and pMHC recognition prediction as the main computational steps. The immunoinformatics tools used for these steps and for the overall prediction of neoantigens are systematically summarized and detailed in this review.

## Introduction

Cancer cells, which form malignant tumors, internally generate aberrant proteins resulting from genetic mutations or other alterations [1]. These proteins are degraded into aberrant peptides, some of which are presented on cancer cell surfaces by major histocompatibility complex (MHC) molecules [1]. When recognized by T cells of the host immune system, these peptides can trigger an immune response aimed at eliminating cancer cells [1]. These presented and recognized peptides are referred to as neoepitopes, and the proteins from which they are derived are called neoantigens. Although neoantigens can generate anticancer immune responses, these responses are insufficient in cancer patients, leading to cancer progression [2]. Neoantigen-based immunotherapies, such as personalized cancer vaccines, are designed to improve the ability of the immune system to target and destroy cancer cells presenting neoantigens [2, 3]. Accurate prediction of neoantigens is critical for the effective design of these therapies [3].

Neoantigen prediction has been previously reviewed from various perspectives [2–7]. For example, Xie *et al.* presented the immunological mechanisms and clinical applications of neoantigens, primarily targeting immunologists and clinicians [2]. Borden *et al.* presented the current knowledge and limitations in neoantigen prediction, prioritization, and validation, targeting readers who already have solid knowledge about neoantigen [3]. Katsikis *et al.* focused on the key obstacles to the applications of neoantigen-based anticancer therapy, targeting scientists performing clinical research [4]. Mattos-Arruda *et al.* discussed the clinical benefits if neoantigen prediction were improved [5]. Fotakis *et al.* and Dhanda *et al.* offered concise overviews of neoantigen prediction, aiming to help scientists without a computational background understand the basics of the computational methods involved [6, 7].

We wrote this review for computational scientists interested in designing novel computational methods to improve neoantigen prediction and/or exploring existing data resources related to neoantigen prediction. This review presents the computational methods and data resources used in neoantigen prediction, describes the relationships between these methods and resources, outlines the current computational challenges, and provides perspectives on emerging challenges that are likely to arise as neoantigen research advances. Adopting a computational perspective, this review provides only a brief overview of the biological mechanisms of neoantigens, as these mechanisms have been extensively covered in previous reviews [2, 3, 5].

## Overview of neoantigen prediction

**Figure 1:**
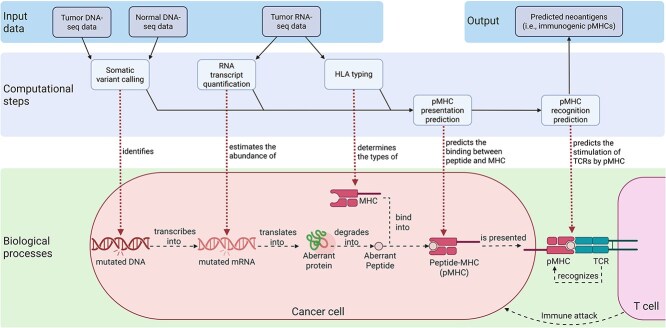
Neoantigen prediction on the basis of the biological mechanism of neoantigen-induced immune attack. The overall prediction pipeline takes tumor DNA-seq, normal (i.e. nontumor) DNA-seq, and tumor RNA-seq data as inputs and outputs the predicted neoantigens. Each computational step in the pipeline corresponds to some neoantigen-related biological processes, which, together, result in T cells attacking cancer cells. Solid arrows are from prerequisite inputs of computational steps to the corresponding outputs. Dashed arrows indicate biological processes. Dotted arrows describe the relationships between the computational steps and the biological processes. This figure was created with Biorender.com. MHC, major histocompatibility complex. TCR, T-cell receptor. Alt text: Neoantigen prediction on the basis of the biological mechanism of neoantigen-induced immune attack.

Neoantigen-induced immune attack operates as a self-reinforcing cycle of cancer cell killing. The process involves the generation, presentation, and recognition of neoantigens, which ultimately triggers the immune response [2, 3, 5] (Fig. 1). In cancer cells, the process begins with somatic DNA mutations, which are transcribed into mutated RNA and translated into aberrant proteins. These proteins are processed into aberrant peptides, which bind to class-I major histocompatibility complex (MHC) molecules, forming peptide–MHC complexes (pMHCs). These pMHCs are presented on the surface of cancer cells and then recognized as nonself by a subset of naive T cells that specifically respond to these pMHCs. This recognition triggers the differentiation of the naive T cells into effector T cells, and the effector T cells proliferate and subsequently destroy cancer cells presenting such pMHCs. The destroyed cancer cells release aberrant peptides, which can be internalized by professional antigen-presenting cells (APCs), such as dendritic cells. Similar to what occurs in cancer cells, within APCs, the internalized peptides bind to class-I MHC molecules to form pMHCs, which are then presented on the APC surface. This presentation further triggers T-cell recognition, amplifying the immune response in a positive feedback loop, resulting in sustained cancer killing.

Some APCs also use class-II MHCs to further strengthen the self-reinforcing cycle of cancer killing [2, 3, 5], but the role of class-II MHCs in cancer immunotherapy remains underexplored [8], with few studies on their potential therapeutic effects [3]. Unless otherwise specified, the term “MHC” refers to class-I MHC in this review. Nonaberrant peptides can also form pMHCs in almost all cells, including noncancer cells, through the same mechanisms. These nonaberrant pMHCs are normally ignored by all T cells due to immune tolerance, thus preventing immune attacks to noncancer cells [2, 3, 5].

The self-reinforcing cancer-killing cycle is too weak in cancer patients, resulting in cancer progression [2]. To strengthen this cycle, we can emulate the release of neoantigens by cancer cells by injecting a large quantity of the neoantigens into the human lymphatic system, in which professional APCs circulate [2, 3]. These APCs can present the neoantigens to the corresponding subset of T cells that recognize these neoantigens. Then, the recognition induces the killing of cancer cells, causing the release of neoantigens from cancer cells to these APCs, thereby strengthening this cancer-killing cycle.

Neoantigens are generated by tumor-specific random mutations (Fig. 1). Thus, neoantigens are patient-specific, requiring prediction from patient-specific sequencing data [3]. On the basis of the biological mechanism of neoantigen-induced immune response, neoantigens can be computationally predicted using specialized pipelines (Fig. 1). A typical pipeline processes raw DNA and RNA sequencing data in FASTQ format and outputs a list of predicted pMHCs corresponding to neoantigens. The pipeline involves several computationally challenging tasks, each of which offers opportunities for optimization to enhance neoantigen prediction performance:

1. Human leukocyte antigen (HLA) typing: Determining the patient’s HLA (i.e. human MHC) allotype, which represents the highly polymorphic alleles of the MHC loci (Fig. 1). Typically, the input and output of this task are RNA-seq data and HLA protein sequences, respectively.
2. RNA transcript quantification: Estimating the abundance of aberrant peptides, on the basis of RNA expression data (Fig. 1). Typically, the input and output of this task are RNA-seq data and transcripts per million (TPM) of each gene transcript, respectively.
3. Somatic variant calling: Identifying cancer-specific somatic variants that generate aberrant peptides (Fig. 1). Typically, the input of this task is tumor and normal whole-exome sequencing (WES) data, and the output of this task is a variant call format (VCF) file that is used to predict aberrant peptides.
4. Peptide–MHC (pMHC) presentation (i.e. binding) prediction: Predicting the binding between aberrant peptides and MHC molecules to generate pMHCs (Fig. 1). The input of this task is typically provided in a tabular format, where each row represents a neoantigen candidate and each column captures specific characteristics, such as the peptide sequence (generated by the somatic variant calling step), MHC type (generated by the HLA typing step), and peptide abundance (estimated by the RNA transcript quantification step). The output of this task is the predicted binding strength of each pMHC.
5. Peptide–MHC (pMHC) recognition prediction: Predicting the likelihood that each pMHC will be recognized by some subset of the T cells of the host immune system (Fig. 1). The input of this task is the same as the pMHC presentation prediction step, except for the addition of an extra column describing the predicted binding strength of pMHC. The output of this task is the predicted recognition strength.

In addition to these tasks, constructing a neoantigen prediction pipeline presents its own computational challenges. This review will briefly cover each task in the context of neoantigen prediction and provide the tools (Tables 1–4), benchmarks (Table 5), and databases (Table 6) used for each task.

**Table 1:** List of computational tools for human leukocyte antigen (HLA) typing

Name	Input data	HLA class	Software availability	Ref.
arcasHLA	WGS, WES, or RNA-seq	I and II	github.com/RabadanLab/arcasHLA	[124]
ATHLATES	WES, WGS^a^, or RNA-seq^a^	I and II	www.broadinstitute.org/viral-genomics/athlates	[125, 126]
Athlon	ONS	I	github.com/cliu32/Athlon	[127]
HISAT-genotype	WGS, WES, or RNA-seq	I and II	daehwankimlab.github.io/hisat-genotype	[11]
HLA*LA	WGS, WES, or LRS	I and II	github.com/DiltheyLab/HLA-LA	[128]
HLA-HD	WGS, WES, or RNA-seq	I and II	w3.genome.med.kyoto-u.ac.jp/HLA-HD	[129–131]
HLA-VBSeq v2.0	WGS or SMRT	I	nagasakilab.csml.org/hla	[132]
HLAFOREST	RNA-seq	I and II	github.com/hyjkim/hlaforest	[133]
HLAminer 1.3.1	WGS, WES, or RNA-seq	I and II	github.com/bcgsc/HLAminer	[134]
HLAminer 1.4	WGS, WES, RNA-seq, amplicon, PacBio, or ONS	I and II	github.com/bcgsc/HLAminer	[135]
HLAreporter	WGS or WES	I and II	paed.hku.hk/genome	[136]
HLAscan	WGS or WES	I and II	github.com/SyntekabioTools/HLAscan	[137]
Kourami	WGS or WES	I and II	github.com/Kingsford-Group/kourami	[10]
OptiType	WGS, WES, or RNA-seq	I	github.com/FRED-2/OptiType	[12]
PolySolver	WES	I	github.com/jason-weirather/hla-polysolver	[13]
seq2HLA	RNA-seq	I and II	github.com/TRON-Bioinformatics/seq2HLA	[138]
SOAPTyping	Sanger sequencing	I and II	github.com/BGI-flexlab/SOAPtyping	[139]
SpecHLA	WGS, WES, RNA-seq, or LRS	I and II	github.com/deepomicslab/SpecHLA	[140]
xHLA	WGS or WES	I and II	github.com/humanlongevity/HLA	[14]

HLA, human leukocyte antigen; WGS, whole-genome sequencing; WES, whole-exome sequencing; ONS, Oxford Nanopore sequencing; LRS, long-read sequencing; SMRT, single-molecule real-time sequencing.

^a^ATHLATES has been tested with only WES but should also be applicable to WGS and RNA-seq data [125, 126].

**Table 2:** List of computational tools for peptide–major histocompatibility complex (pMHC) binding-strength prediction

Name	Core algorithm	Output	HLA class	Software availability	Ref.
ACME	Attention-based CNN	BA	I	github.com/HYsxe/ACME	[141]^a^
BERTMHC	BERT and MIL	EL and BA	II	github.com/s6juncheng/BERTMHC	[142]
BigMHC	TL	EL, BA, and IG	I	github.com/KarchinLab/bigmhc	[143]
CapsNet-MHC	Capsule network	BA	I	github.com/s7776d/CapsNet-MHC	[144]
ConvMHC	ANN	BA	I	github.com/aidanbio/convmhc	[145]^a^
DeepAttentionPan	CNN and AM	BA	I	github.com/jjin49/DeepAttentionPan	[146]
DeepMHCI	CNN	BA	I	github.com/ZhuLab-Fudan/DeepMHCI	[147]
DeepMHCII	CNN	BA	II	github.com/ZhuLab-Fudan/DeepMHCII	[147]
DeepSeqPan	Interpretable RNN and AM	BA	I	github.com/pcpLiu/DeepSeqPan	[148]
DeepSeqPanII	Interpretable RNN and AM	BA	II	github.com/pcpLiu/DeepSeqPanII	[149]
ForestMHC	ANN	EL	I	github.com/kmboehm/ForestMHC	[150]^a^
Graph-pMHC	GNN and Alphafold2 [151]	EL	II	github.com/Genentech/gpmhc	[152]
HLAthena	ANN	EL	I	hlathena.tools	[153]
IEDB SMM	SMM	BA	I	github.com/aidanbio/convmhc	[154]^a^
MARIA	ANN	EL	II	maria.stanford.edu/about.php	[155]
MHCflurry2.0	ANN	EL and BA	I and II	github.com/openvax/mhcflurry	[51]^a^
MHCnuggets	LSTM	BA	I and II	github.com/KarchinLab/mhcnuggets	[156]^a^
MHCSeqNet	RNN	EL	I	github.com/cmb-chula/MHCSeqNet	[157]
MHCSeqNet2	Structure embedding	BA	I	github.com/cmb-chula/MHCSeqNet2	[158]
MixMHC2pred	Structure-based ML	EL	II	github.com/GfellerLab/MixMHC2pred	[52]
MixMHCpred	Matrix approach	EL	I	github.com/GfellerLab/MixMHCpred	[159]^a^
MSIntrinsic	ANN	EL	I	N/A	[160]^a^
NetMHC	ANN	BA	I	services.healthtech.dtu.dk/service.php?NetMHC-4.0	[161]
NetMHC4.0	ANN	BA	I	services.healthtech.dtu.dk/services/NetMHC-4.0	[162]^a^
NetMHCcons	Consensus	BA	I	www.cbs.dtu.dk/services/NetMHCcons	[163]
NetMHCIIpan-4.0	ANN	EL and BA	II	services.healthtech.dtu.dk/services/NetMHCIIpan-4.0	[50]
NetMHCpan4.1	ANN	EL and BA	I and II	services.healthtech.dtu.dk/services/NetMHCpan	[50]^a^
NNAlign	ANN	EL and BA	I and II	services.healthtech.dtu.dk/services/NNAlign-2.1	[164]^a^
PAComplex	Structural modeling	Binding model	I	http://PAcomplex.life.nctu.edu.tw (no longer available)	[165]^a^
PickPocket	SMM	BA	I	www.cbs.dtu.dk/services/PickPocket	[166]^a^
PRIME	GLM	EL	I	github.com/GfellerLab/PRIME	[53]
PUFFIN	EnsL and DRN	BL	I	https://github.com/gifford-lab/PUFFIN	[167]
PUFFIN	EnsL and DRN	BL	II	github.com/gifford-lab/MHC2-optimization	[168]
RBM-MHC	RBM	APP	I	github.com/bravib/rbm-mhc	[169]
RPEMHC	CNN and LSTM	BA	I and II	github.com/lennylv/RPEMHC	[170]
SMMPMBEC	SMM	BA	I	github.com/ykimbiology/smmpmbec	[171]^a^
STMHCpan	Star-Transformer	APP	I	github.com/Luckysoutheast/STMHCPan	[172]
SYFPEITHI	Motifs	BA	I and II	www.syfpeithi.de	[65]^a^
TLimmuno2	LSTM and TL	BA and immunogenicity	II	https://github.com/XSLiuLab/TLimmuno2	[173]
TripHLApan	TCM, BiGRU, AM, and TL	BP	I and II	github.com/CSUBioGroup/TripHLApan	[174]
USMPep	RNN	BA	I and II	github.com/nstrodt/USMPep	[175]

AM, attention mechanism; ANN, artificial neural network; APP, antigen-presentation probability; BA, binding affinity; BL, binding likelihood (including uncertainty in its estimation); BP, binding probability; BERT, Bidirectional Encoder Representations from Transformer; CNN, convoluted neural network; DCNN, deep convoluted neural network; DRN, deep residual network; EL, eluted-ligand likelihood; EnsL, ensemble learning; GNN, graph neural network; GBDT, gradient-boosted decision trees; GLM, generalized linear model; HLA, human leukocyte antigen; IG, immunogenicity; LSTM, long short-term memory; MIL, multiple-instance learning; ML, machine learning; RBM, restricted Boltzmann machine; RNN, recurrent neural network; SMM, stabilized matrix method; TCM, triple coding matrix; TL, transfer learning.

^a^Adapted from Borden *et al.* [3] licensed under the CC BY 4.0 license © 2022.

**Table 3:** List of computational tools to predict the interactions between peptide–major histocompatibility complexes (pMHCs) and T-cell receptors (TCRs)

Name	TCR chain input	Training data	Core algorithm(s)	Software availability	Ref.
CATCR	Single	[176]	Convolutional-self-AM	github.com/FreudDolce/CTTCR	[176]
DePTH	Single	[177, 178] and McPAS-TCR [71]	CNN and dense layers	github.com/Sun-lab/DePTH	[179]
epiTCR	Single	IEDB [70], TBAdb [180], VDJdb [72], McPAS-TCR [71], and 10× [181]	Random forest and BLOSUM62 encoding	github.com/ddiem-ri-4D/epiTCR	[82]
EPIC-TRACE	Single or paired	IEDB [70] and VDJdb [72]	ProtBERT [182], CNN, and multi-head AM	github.com/DaniTheOrange/EPIC-TRACE	[183]
HeteroTCR	Single	IEDB [70], VDJdb [72], and McPAS-TCR [71]	Heterogeneous GNN	github.com/yuzilan/HeteroTCR	[184]
MIX-TPI	Single or paired	Data use by TITAN [185], ImRex [186], and NetTCR-2.0 [187]	CNN, multi-modality, and self-AM	github.com/GfellerLab/MixTCRpred	[188]
MixTCRpred	Paired	IEDB [110], McPas-TCR [107], VDJdb [111], 10× [124], and multiple public sources (see [189] for detail)	Transformer	github.com/GfellerLab/MixTCRpred	[189]
NetTCR 2.2	Paired	Described in [84 Training data]	Trial-and-error with many ML techniques	github.com/mnielLab/NetTCR-2.2	[84]
PanPep	Single	IEDB [110], McPas-TCR [71], PIRD [180], VDJdb [111], and the control TCR set (see [85] for detail)	Meta-learning and neural Turing machine	github.com/bm2-lab/PanPep	[85]
POP-UP TCR	Single or paired	McPas-TCR [71]	ML approach with two stages	github.com/NiliTicko/POP-UP-TCR	[190]
STABLER	Paired	[192 Extended Data Fig. 1]	BERT	github.com/NKI-AI/STAPLER	[191]
TABR-BERT	Single	zenodo.org/records/8215354	BERT	github.com/Freshwind-Bioinformatics/TABR-BERT	[192]
TCR-ESM	Single or paired	IEDB [70], VDJdb [72], and 10× [181]	ESM1v [193]	gitHub.com/dhanjal-lab/tcr-esm	[67]
TCR-Pred	Single	VDJdb [72], McPAS-TCR [71], and IEDB [70]	SAR using MNA with MultiPASS [194]	way2drug.com/TCR-pred (no longer available)	[195]
TCRmodel2	Single	N/A (because the software performs structure modeling)	AlphaFold2 [151]	github.com/piercelab/tcrmodel2	[67]
VitTCR	Single	IEDB [70] and McPAS-TCR [71]	ViT	github.com/Jiang-Mengnan/VitTCR	[67]

Such tools that were published between 03/2020 and 08/2022 (e.g. ERGO-II) have been listed in [77 Table 1] and are therefore not shown here. AM, attention mechanism; BERT, bidirectional encoder representations from transformers; CNN, convolutional neural network; ESM, evolutionary scale modeling; GNN, graph neural network; IEDB, Immune Epitope Database; ImRex, interaction map recognition; McPas-TCR, manually curated catalogue of pathology-associated TCR sequences; ML, machine learning; MNA, multilevel neighborhoods of atoms; SAR, structure-activity relationships; TCR, T cell receptor; TITAN, TCR epitope bimodal attention networks; VDJdb, VDJ database; ViT, vision transformer.

**Table 4:** List of bioinformatics pipelines for neoantigen prioritization

Name	Input(s)^a^	Output	HLA class	Neoantigen source(s)	Software availability	Ref.
Antigen.garnish	VCF, RNA sequence, or peptide sequence	Features	I and II	SNV, InDel, and GF	github.com/andrewrech/antigen.garnish	[73]
CloudNeo	VCF, and DNA-seq or RNA-seq BAM	Features	I	N/A	github.com/TheJacksonLaboratory/CloudNeo	[196]^a^
DeepAntigen	VCF	Features & ranks	I	SNV and InDel	yishi.sjtu.edu.cn/deepAntigen	[197]
DeepHLApan	CSV specifying peptide-HLA pairs	Features & ranks	I	N/A	github.com/jiujiezz/deephlapan	[24]^a^
Epidisco	WES and RNA-seq FASTQ	Features & ranks (using VaxRank [96])	I	SNV, InDel, GF, and SpV	github.com/hammerlab/epidisco	[198]
epitopeprediction	Mutation VCF	Features	I and II	SNV and InDel	github.com/nf-core/epitopeprediction	[199]
Fred2	VCF	Features	I	N/A	github.com/FRED-2/Fred2	[200]^a^
ImmuneMirror	WES and RNA-seq FASTQ or VCF	Features & ranks	I and II	SNV and InDel	github.com/weidai2/ImmuneMirror	[201]
INeo-Epp	Peptide FASTA and HLA alleles	Features & ranks	I	N/A	www.biostatistics.online/INeo-Epp/antigen.php	[202]^a^
INTEGRATE-neo	HLA alleles^b^ and INTEGRATE output [203]	Features	I	GF	github.com/ChrisMaherLab/INTEGRATE-Neo	[204]^a^
MuPeXi	VCF, HLA alleles, and optionally gene expression	Features & ranks	I	N/A	services.healthtech.dtu.dk/services/MuPeXI-1.1	[23]^a^
neoANT-HILL	VCF, and/or RNA-seq BAM or FASTQ	Features	I	SNV and InDel	github.com/neoanthill/neoANT-HILL	[205]^a^
neoantigenR	DNA-seq FASTQ, RNA-seq FASTQ, and GFF	Features	I	SNV and InDel	github.com/tangshao2016/neoantigenR	[206]^a^
neoantigenR	DNA-seq FASTQ, RNA-seq FASTQ, and GFF	Features	I	SNV and InDel	github.com/tangshao2016/neoantigenR	[206]^a^
Neoantimon	VCF or mutant RNA sequences, and HLA alleles	Features	I and II	SNV, InDel, and SV	github.com/hase62/Neoantimon	[207]
Neoepiscope	VCF, DNA-seq BAM, and optionally HLA alleles	Features	I and II	SNV and InDel	github.com/pdxgx/neoepiscope/nf-core	[208]^a^
NeoepitopePred	WGS and RNA-seq FASTQ or BAM	Features	I	SNV and GF	github.com/XingTang2014/NeoepitopePred^c^	[209]^a^
NeoFlow	VCF, DNA-seq or RNA-seq FASTQ, and MS MGF	Features	I	All^c^	github.com/bzhanglab/neoflow	[210]
NeoFox	Peptides, HLA alleles, and optionally other properties	Features	I and II	N/A	github.com/TRON-Bioinformatics/neofox	[211]
NeoFuse	RNA-seq FASTQ	Features & ranks	I and II	GF	github.com/icbi-lab/NeoFuse	[212]
NeoHunter	VCF, or WES and RNA-seq FASTQ	Features & ranks	I	SNV, InDel, GF, and SpV	github.com/XuegongLab/NeoHunter	[75]
Neopepsee	RNA-seq FASTQ, VCF, and optionally HLA alleles	Features & ranks	I	SNV	sourceforge.net/p/neopepsee/wiki/Home	[213]^a^
NeoPredPipe	VCF and optionally HLA alleles	Features & ranks	I and II	SNV and InDel	github.com/MathOnco/NeoPredPipe	[214]^a^
NextNEOpi	WES or WGS, and optionally RNA-seq, FASTQ or BAM	Features	I and II	SNV, InDel, and GF	github.com/icbi-lab/nextNEOpi	[215]
OpenVax	WES and RNA-seq FASTQ	Features & ranks (using VaxRank [96])	I	SNV	github.com/openvax/neoantigen-vaccine-pipeline	[97]
PGNneo	RNA-seq FASTQ and MS RAW	Features	I	All^c^	github.com/tanxiaoxiu/PGNneo	[216]
ProGeo-neo	VCF and RNA-seq FASTQ	Features	I	SNV	github.com/kbvstmd/ProGeo-neo	[217]^a^
ProGeo-neo-2	DNA-seq FASTQ, RNA-seq FASTQ, and MS RAW	Features	I and II	All^c^	github.com/kbvstmd/ProGeo-neo-2	[218]
ProTECT	DNA-seq and RNA-seq FASTQ or BAM, and/or VCF	Features & ranks	I and II	SNV	github.com/BD2KGenomics/protect	[219]
pTuneos	(1) WES and RNA-seq FASTQ, or (2) VCF, gene-expression, and copy-number profiles	Features & ranks^d^	I	SNV and InDel	github.com/bm2-lab/pTuneos	[74]^a^
pVACtools^e^	Peptide FASTA, VCF, and HLA alleles, AGFusion [220] results (or Arriba [221] TSV), StarFusion [222] TSV	Features & tiers	I and II	N/A	github.com/griffithlab/pVACtools	[95]^a^
ScanNeo	RNA-seq BAM	Features & ranks	I	InDel	github.com/ylab-hi/ScanNeo	[223]^a^
ScanNeo2	RNA-seq and optionally DNA-seq BAM	Features & rank	I and II	InDel, GF, and SpV	github.com/ylab-hi/ScanNeo2	[224]
Seq2Neo	WES (and optionally RNA-seq) FASTQ or VCF	Features & ranks	I	SNV, InDel, and GF	github.com/XSLiuLab/Seq2Neo	[225]
TepiTool	Peptide FASTA and HLA alleles	Features	I and II	N/A	tools.iedb.org/tepitool	[226]^a^
TIminer	RNA-seq FASTQ and VCF	Features	I	SNV	icbi.i-med.ac.at/ software/timiner/timiner.shtml	[227]^a^
TruNeo	WES and RNA-seq FASTQ	Features	I	SNVs and InDel	github.com/yucebio/TruNeo	[228]
TSNAD v2.0	WES and RNA-seq FASTQ	Features & ranks	I	SNV and InDel	github.com/jiujiezz/tsnad	[99]^a^
VaxRank^f^	VCF, RNA-seq FASTQ, and HLA alleles	Features & ranks	I	N/A	github.com/openvax/vaxrank	[96]^a^

DNA-seq (including WES and WGS) input contains tumor-normal paired sequencing data, RNA-seq and MS inputs contain tumor-only data, and VCF input contains somatic variants. The output can be the features of candidate neoantigens and/or the ranks (or tiers) of their therapeutic potentials. BAM, binary alignment/map; GF, gene fusion; InDel, insertion-deletion; N/A: not applicable; MS, mass spectrometry; SNV, single-nucleotide variant; SpV, splicing variant; SV, structural variant; TSV, tab-separated version; VCF, variant-call format; WES, whole-exome sequencing; WGS, whole-genome sequencing.

^a^Adapted from Gopanenko, *et al.* [229] licensed under the CC BY 4.0 license © 2018.

^b^The originally published link github.com/zhanglabstjude/neoepitope no longer works.

^c^Some neoepitopes are detected from mass spectrometry as amino acid sequences, so the genomic and/or transcriptomic origins of the neoepitopes can be unknown.

^d^Sorted by a combination of recognition score and intra-tumor heterogenicity statistics.

^e^The clinical trial NCT02348320 [230] used pVACtools.

^f^The clinical trials NCT02721043 [231], NCT03223103 [232], and NCT03359239 [233] used VaxRank.

## Human leukocyte antigen typing

HLA typing is the process of determining the patient’s HLA allotype, which represents the highly polymorphic alleles encoded by the MHC of humans. This process is a critical component of neoantigen prediction, as HLA molecules of different allotypes bind to peptides with distinct amino acid sequences. Accurate HLA typing is therefore a prerequisite for predicting the formation of pMHCs.

Owing to the importance of HLA typing, several computational methods have been developed [9]. For example, a tool named Kourami infers the most likely path in a graph of reads aligned to the reference and uses this path to represent the HLA allotype, and such inference can discover novel HLA alleles but requires high-coverage whole-genome sequencing (WGS) data [10]. HISAT-genotype uses graph-based alignment (HISAT2) with an expectation–maximization algorithm, and this approach is accurate but computationally intensive [11]. OptiType uses integer linear programming to identify the HLA allele combination that best explains the sequencing reads [12], and this approach runs fast but is unable to discover novel HLA alleles. The HLA typing tools typically use patient data generated by WGS, WES, and/or RNA-seq (Table 1). Commonly used tools for HLA typing include OptiType [12], HISAT-genotype [11], PolySolver [13], and xHLA [14].

Previous benchmarks showed that the state-of-the-art methods for HLA typing have demonstrated high performance (Table 5). Nevertheless, HLA typing is subject to certain considerations. RNA-seq is preferred over DNA-seq for HLA typing whenever possible, as it can detect allele downregulation at the RNA level that DNA-seq cannot. DNA methylation, an epigenetic modification, may reduce the expression of specific HLA alleles, which can be revealed by RNA-seq [15].

The specifications of the HLA allotypes used for HLA typing were derived from the Immuno-Polymorphism Database of the international ImMunoGeneTics project for human leucocyte antigens (IPD-IMGT/HLA), which provides sequences of human MHC molecules and their official names designated by the World Health Organization Nomenclature Committee for Factors of the HLA System [16]. The database also provides a searchable repository of highly curated HLA sequences. An extension, IPD-MHC, expands this resource to include MHC data for both human and nonhuman species [17]. These resources are critical for developing HLA typing methods.

## RNA transcript quantification

Neoantigen abundance refers to the quantity of a specific neoantigen expressed by cancer cells and is typically represented by the quantity of RNA transcripts encoding the neoantigen. RNA transcript quantification is a critical part of neoantigen prediction, as the abundance of RNA transcripts encoding a potential neoantigen is positively correlated with the immunogenicity of the neoantigen [18]. In the context of neoantigen prediction, immunogenicity refers to the ability to provoke an immune response against cancer cells.

The first-generation computational methods for RNA-transcript quantification, such as RNA-Seq by Expectation Maximization (RSEM), assign reads to RNA transcripts to maximize the likelihood of observing the sequencing data given the assignment (e.g. by using the expectation–maximization algorithm) [19]. To reduce the running time of the likelihood-based methods without significantly compromising accuracy, tools such as Kallisto and Salmon employ pseudoalignment [20]. Commonly used tools for RNA transcript quantification, in the context of neoantigen prediction, include Kallisto [20], RSEM [21], and Salmon [22].

Previously published benchmarks indicate high performance among these methods (Table 5). Despite their accuracy, transcript quantification methods have limitations within the neoantigen prediction pipeline. For example, some studies may not provide the RNA-seq data used for transcript quantification [23, 24]. In such cases, the pipeline should operate seamlessly without RNA-seq input. Additionally, mass spectrometry (MS) can reveal neoantigen abundance by directly measuring the amount of each neopeptide (i.e. neoantigen-derived peptide) in tumors [25], but its low sensitivity limits its effectiveness for neoantigen detection [25, 26].

When RNA-seq data are unavailable, gene expression (i.e. neoantigen abundance at the RNA level) can be estimated using databases. Resources such as The Cancer Genome Atlas (TCGA) [27], Gene Expression Nebulas [28], the Gene Expression Omnibus (GEO) [29], GeneCards [30], GTEx [31], and hECA [32] provide comprehensive gene expression datasets that can serve as substitutes for RNA-seq analysis results in these scenarios. These resources supplement neoantigen prediction pipelines when RNA-seq data are lacking.

## Somatic variant calling

Somatic variant calling refers to the identification of tumor-specific variants generated by somatic mutations harbored by cancer cells. The variants used for neoantigen prediction often include single-nucleotide variants (SNVs) and insertion–deletions (InDels). Variant calling is an integral part of neoantigen prediction, as some somatic variants give rise to aberrant peptides that act as neoepitopes (Fig. 1). Unless explicitly stated otherwise, “variant call” in this context refers to “somatic variant call.”

Variant callers are bioinformatics tools designed to identify genetic variants from sequencing data [33]. Such a tool generally takes sequencing data in FASTQ or BAM format as input and produces a VCF file as output. A variant caller should both minimize false-positive calls and maximize true-positive calls. To achieve this, variant callers often employ approaches such as heuristic thresholds, statistical testing, allele fraction (AF) analysis, haplotype-based strategies, and/or machine learning [34]. Almost all variant callers internally use heuristic thresholds, statistical testing, and AF analysis. Some variant callers use the haplotype-based strategies to improve performance in repetitive genome regions [35] at the cost of consuming extra computational resources [36] and/or machine learning to improve performance at the cost of requiring training data and extra computation time. Commonly used tools for somatic variant calling include VarScan2 [37], Mutect2 [38], and Strelka2 [39]. Some germline variant callers, such as FreeBayes, can also call somatic variants by using custom-designed command-line parameters, although germline variant callers are inherently unable to compare the tumor sample with the matched normal sample [35, 40].

The state-of-the-art methods for calling variants that generate highly clonal neoantigens achieved medium performance, as highlighted in published benchmarks (Table 5). A neoantigen is highly clonal if it is presented by a large proportion of cancer cells, and this proportion linearly correlates with the variant AF (VAF) of the mutation generating the neoantigen. Typically, for VAF values above 0.05, the precision and recall rates for variant calling exceed 0.95, provided that the BAM file used for variant calling is generated by a standard next-generation sequencing (NGS) aligner such as Burrows-Wheeler Aligner with Maximal Exact Matches (i.e. BWA MEM) [41].

While current tools perform well for high-clonality neoantigens, their performance depends on factors such as sample type, assay type, tumor purity, normal purity, and sequencing depth [36]. Thus, these factors should be carefully considered in variant calling. Additionally, we can use these factors, as well as unforeseeable anomalies (e.g. abnormally low sequencing depth at the variant locus), to filter out false-positive variant calls. Thus, in clinical settings, the variants identified by variant callers are usually reviewed by human experts using tools such as the Integrated Genomic Browser, a tool for manually reviewing these variants [42]. Usually, the identified variants are subsequently annotated via programs such as ANNOtate VARiation (i.e. ANNOVAR) [43] or Variant Effect Predictor (i.e. VEP) [44] to eliminate those that cannot produce aberrant peptides.

**Table 5:** Previously published performances of neoantigen prediction tasks

Neoantigen-prediction task	Benchmark results
HLA typing	OptiType, HiSAT-genotype, and HLA-HD have accuracies of 99%, 100%, and 100% for typing HLA alleles at four-digit resolution^a^, respectively [9, 234]. Optitype has a typing accuracy above 95% for all HLA loci [235].
RNA transcript quantification	Kallisto has a multiplicative error rate of 5% [20]. Correlation between the RNA expression measured by various tools and TaqMan RT-PCR is between 0.85 and 0.90 [236]. Most tools can achieve a Kendall’s tau b correlation of 0.85 between the observed and expected RNA expression levels [237].
Somatic variant calling	Precision and recall both surpass 0.95 if the VAF is at least 0.05 for cancer-related variants [41]. F-scores are 0.9 and 0.8 for SNV and InDel variants [238].
pMHC presentation prediction	AUROC is generally above 0.85 when using the experimentally measured IC50 threshold of 500 nM to partition the peptide-HLA pairs into positives and negatives [239–241]. Accuracy, sensitivity, specificity, MCC, and AUROC are generally all above 0.95 when using MS-derived, MHC-eluted ligand assay as the ground-truth [49, 240].
pMHC recognition prediction	The AUROC is approximately 50% for TCR-specific prediction [78, 242].
Overall neoantigen prediction	Less than half of immunogenic peptides are found in the top-20 ranked peptide candidates [18, 98].

AUROC, area under the receiver operating characteristic curve; HLA, human leucocyte antigen; MCC, Matthews correlation coefficient; MS, mass spectrometry; pMHC, peptide-bound MHC; VAF, variant allele fraction.

^a^Two HLA allotypes that are identical at four-digit resolution generate HLA proteins with the same amino acid sequences.

**Table 6:** A catalog of the databases mentioned in this review

Neoantigen-prediction task	Relevant database	Characteristics and utility	Link	Ref.
HLA typing	IMGT	Provides official HLA sequences and names	https://www.imgt.org/	[16]
	IPD-MHC	Expands IMGT to include both human and nonhuman species	https://www.ebi.ac.uk/ipd/mhc/	[17]
RNA transcript quantification	TCGA^a^	Provide gene expression data	https://portal.gdc.cancer.gov/	[27]
	GEO		https://www.ncbi.nlm.nih.gov/geo/	[28]
	GeneCards		https://www.genecards.org/	[29]
	GTEx		https://www.gtexportal.org/	[30]
	hECA		https://eca.xglab.tech/	[31]
Somatic variant calling	dbSNP	Provides commonly observed human genetic variations to filter out germline variants	https://www.ncbi.nlm.nih.gov/snp/	[45]
	1000 Genomes Project		https://www.internationalgenome.org/	[46]
	COSMIC	Provides somatic mutations to filter out germline variants	https://cancer.sanger.ac.uk/cosmic/	[47]
pMHC presentation prediction	SYFPEITHI	Provides MHC class I and class II ligands and peptide motifs	http://www.syfpeithi.de/	[65]
	DFRMLI		http://bio.dfci.harvard.edu/DFRMLI (no longer available)	[66]
	DFRMLI	Provides experimentally validated T-cell epitopes derived from tumor or virus antigen		[66]
pMHC recognition prediction	IEDB	Provides experimental data on antibodies and T-cell epitopes	https://www.iedb.org/	[70]
	CEDAR	Provide the cancer-specific data of IEDB	https://cedar.iedb.org/	[88]
	ImMunoGeneTics	Provides popular databases and tools in the fields of immunogenetics and immunoinformatics	https://www.imgt.org/	[90]
	VDJdb	Provides TCR and antigen sequences such that the TCR recognizes the antigen	https://vdjdb.cdr3.net/	[72]
	McPAS-TCR		https://friedmanlab.weizmann.ac.il/McPAS-TCR/	[71]
	ATLAS	Provides TCR structures and interactions between pMHCs and TCRs	https://atlas.wenglab.org/web/index.php	[91]
	TCR3d		https://tcr3d.ibbr.umd.edu/	[92]

HLA, human leucocyte antigen; MHC, major-histocompatibility complex; pMHC, peptide-bound MHC; TCR, T-cell receptor.

^a^We can use TCGA for additional analyses to improve the characterization of neoantigens.

Variant databases can increase the accuracy of somatic variant calling by providing reference data for eliminating or retaining relevant variants. The dbSNP database provides a comprehensive collection of commonly observed human genetic variations [45], whereas the 1000 Genomes Project database provides the frequencies of very commonly observed genetic variations in a population of ~1000 human individuals [46]. Both databases are typically used to filter out germline variants, which exist in both cancer and noncancer cells. Variants occurring in at least 5% of human individuals are typically considered to be of germline origin and are thus filtered out. The Catalogue Of Somatic Mutation In Cancer (COSMIC) database contains information on somatic mutations and their occurrence frequencies in cancer patients [47]. The COSMIC database allows the retention of somatic variants specific to cancer cells. Variants occurring at least five times in the COSMIC database are highly likely to be of somatic origin and hence should be retained, even if they would otherwise be filtered out.

## Peptide–major histocompatibility complex presentation prediction

After the prediction of HLA allotypes and aberrant peptides, the subsequent step is to determine which HLA molecule binds to which aberrant peptide to form a pMHC, which is presented to the cancer cell surface. The determination of this binding strength can result from the estimation of pMHC binding affinity, which is inversely proportional to the half-maximal inhibitory concentration (IC50) typically measured in nanomolar concentrations (nM). Accurate pMHC binding affinity prediction is essential for neoantigen prediction, as peptides and MHCs individually are not recognized by the immune system—only pMHCs can potentially trigger a response (Fig. 1). A high binding affinity (i.e. low IC50) between a peptide and an MHC increases the likelihood that the peptide will bind to form a pMHC, making its accurate prediction critical for identifying immunogenic neoantigen candidates.

The methods for predicting pMHC binding affinity usually use scoring, neural network, and/or consensus-based approaches (Table 2) [48]. Scoring-based methods integrate features using statistical scoring functions, such as Markov models, position-specific scoring matrices, and BLOSUM 62 matrices, to calculate a likelihood that correlates with pMHC binding affinity [49]. Neural network–based methods train models on datasets of known peptide–MHC binding pairs with experimentally measured affinities, enabling the prediction of unknown pMHCs [49]. Consensus-based methods employ ensemble learning to combine predictions from multiple methods [49]. Commonly used tools for pMHC binding strength prediction include NetMHCpan4.1 [50], MHCflurry2.0 [51], MixMHC2pred [52], and PRIME [53], which are all based on neural networks (tools such as NetMHCpan4.1 and MHCflurry2.0 also internally use the consensus of the predictions provided by multiple neural networks). Neural network–based methods tend to outperform scoring-based methods (Table 5).

The state-of-the-art methods for pMHC binding affinity prediction achieved medium performance, as shown by previous benchmarks (Table 5). However, the performance varies with the HLA allotype of interest [54]. For example, estimating the pMHC binding affinities of the HLA-A02:01 allotype is more challenging than those of other HLA allotypes [49]. While sequence-based predictors remain the gold standard, structure-based predictors can still correct some erroneous estimations made by sequence-based predictors [55–58].

In addition to binding affinity, pMHC elution-ligand likelihood can also represent the binding strength between the peptide and the MHC that together form the pMHC. MS enables direct observations of an ensemble of MHC-bound peptides (i.e. the immunopeptidome) from MHC ligand elution [25]. Owing to the high throughput of MS, the combination of MHC ligand elution and MS provides abundant training data for machine learning algorithms that predict the formation of MHC-bound peptides [59]. Furthermore, MHC ligand elution intrinsically considers the entire biological process of pMHC presentation, whereas pMHC binding affinity is only a single biochemical property [60]. Consequently, the use of MHC ligand elution with MS is becoming increasingly popular [61]. For the task of predicting MS-derived and MHC-eluted ligands, the accuracy, sensitivity, specificity, Matthews correlation coefficient, and area under the receiver operating characteristic curve (AUROC) are generally above 0.95 (Table 5).

pMHC binding stability also positively correlates with the strength of the interaction between the MHC molecule and the peptide. The binding stability of a pMHC is the expected elapsed time, typically measured in hours, before the dissociation of the pMHC into its constituent peptide and MHC. The more stable a pMHC complex is, the longer it spends its time attempting to generate an immune response, and the strength of the response potentially increases superlinearly with respect to the time spent [62]. Unlike pMHC binding affinity, labeled data with experimentally measured ground truths are scarce for predicting binding stability [63]. To the best of our knowledge, netMHCstabpan 1.0 is currently the only software package for estimating binding stability [64]. More labeled data and software packages are needed to improve binding stability estimation.

The key resources for pMHC presentation studies include the MHC databank SYFPEITHI [65] and the Dana-Farber repository for machine learning in immunology (DFRMLI) [66]. SYFPEITHI offers a collection of MHC class I and class II ligands and peptide motifs from humans and other species. All entries in SYFPEITHI are compiled from published reports. DFRMLI provides standardized datasets of MHC-binding peptides, with all binding affinities mapped onto a common scale [66]. Both SYFPEITHI and DFRMLI also provide MS-generated immunopeptidomic data, which reveal the repertoire of peptides that bind to MHC molecules.

## Peptide–major histocompatibility complex recognition prediction

The recognition of a presented pMHC refers to the identification of the pMHC as a foreign (nonself) substance by the T-cell receptors (TCRs) of the host immune system. This recognition activates T cells, triggering their proliferation and the subsequent destruction of cancer cells presenting the identified pMHCs. The prediction of pMHC recognition is essential for neoantigen prediction because not all presented pMHCs elicit immune responses.

Each method for predicting pMHC recognition is either TCR-specific or TCR-unspecific. The TCR-specific approach predicts the interaction between each TCR and each pMHC, often using protein sequence embeddings and neural networks, such as convolutional neural networks or long short-term memory networks [67–69]. The tools are typically trained on databases such as the Immune Epitope Database (IEDB) [70], McPAS-TCR [71], and VDJdb [72], which contain data describing experimentally validated TCR–pMHC interactions (Table 3). These tools are typically applicable to only single (i.e. TCR-$\beta$) chain data because data with paired (i.e. both TCR-$\alpha$ and TCR-$\beta$) chains are scarce, as TCR-$\alpha$ chains are less diverse and hence sequenced less often than TCR-$\beta$ chains.

The TCR-unspecific approach, by contrast, considers essential TCR recognition features, such as agretopicity and foreignness, and applies simple rules derived from the biological mechanism of immunogenicity to these features [18]. The agretopicity of a pMHC with a mutant peptide is defined and computed as the ratio of the pMHC binding affinity of the mutant peptide to that of the corresponding wild-type peptide [18]. The foreignness of a peptide is defined as the probability that a random T-cell recognizes the peptide. Foreignness is computed in a manner analogous to the approaches implemented in the antigen.garnish R package [18, 73] and the pTuneo software package [74].

The state-of-the-art methods for pMHC recognition prediction achieved low performance, as shown by previously published benchmarks (Table 5). The TCR-specific approach, despite its use of additional information, performs much worse than the TCR-unspecific approach in neoantigen prediction. This is likely due to the inability of typical sequencing assays to capture the vast diversity of TCR clonotypes [75] and the inherent complexity of predicting TCR–pMHC interactions [76, 77]. The AUROC of current methods barely exceeds 0.5 for predicting whether a TCR clonotype recognizes a pMHC when the training data lack the peptide of the pMHC, indicating that these methods perform only slightly better than random guessing for predicting neoepitopes with peptides absent from the training set [78]. Unfortunately, in clinical settings, such neoepitopes constitute the majority of all neoepitopes [79]. The immense diversity of TCR clonotypes, estimated at ~10 billion in the human body, further complicates the task, as experimentally obtaining all TCR clonotypes for a given patient is impractical [80]. While the TCR-specific approach does not perform well, it remains valuable for studying the biological mechanism of immunogenicity. Consequently, various tools have been developed to predict interactions between pMHCs and TCRs (Table 3). Commonly used tools for predicting these interactions include DLpTCR [81], epiTCR [82], ERGO-II [83], NetTCR 2.2 [84], PanPep [85], pMTNet [86], and TCRmodel2 [67]. As mentioned before, the current TCR-specific tools only perform slightly better than a random guess in the context of neoantigen prediction. Thus, the methodological advantages and disadvantages of each tool remain unknown. The underperformance is caused by the lack of both training data and biological knowledge about pMHC-TCR interaction: we can use only ~1 million pMHC-TCR pairs, most of which contain incomplete TCR sequences, to train a model that tries to predict the effect of changing a single amino acid to the peptide, MHC, or TCR [70–72, 77, 87].

Owing to the current underperformance of the TCR-specific approach, we recommend the TCR-unspecific approach. This approach typically uses agretopicity and foreignness as two key features [18]. The use of these two features improves precision by ~50% while maintaining the same sensitivity in a discovery cohort (i.e. training) dataset consisting of five patients with 34 immunogenic neoepitopes [18].

Several data resources can support the creation of pMHC recognition prediction methods. The IEDB provides experimental data on antibodies and T-cell epitopes (i.e. antigenic determinants, the parts of an antigen that are recognized by the immune system) studied in humans and other animal species, focusing primarily on infectious diseases, allergies, autoimmunity, and transplantation [70]. The Cancer Epitope Database and Analysis Resource (CEDAR) offers experimental data on antibodies and T-cell epitopes, with a primary focus on cancer [88]. The CEDAR also serves as a companion website to the IEDB [88]. Both the IEDB and the CEDAR contain multiple types of immunological data related to pMHC binding, pMHC immunogenicity, and interactions between pMHCs and TCRs [70, 88]. The Immune Epitope Database Analysis Resource (IEDB-AR) is a companion website to the IEDB and offers computational tools that predict and analyze B-cell and T-cell epitopes [89]. Examples of these tools include netMHCpan and netMHCstabpan. Additionally, the international ImMunoGeneTics information system provides popular databases and tools in the fields of immunogenetics and immunoinformatics [90].

In addition to the IEDB, several other databases can be used to study pMHC recognition. DFRMLI offers MHC-binding datasets and a catalog of experimentally validated and naturally processed T-cell epitopes derived from tumor or virus antigens [66]. VDJdb serves as a curated database of TCR sequences known to induce immunogenicity when combined with specific antigens, offering insights into TCR recognition within specific MHC contexts [72]. McPAS-TCR is a manually curated database of TCR sequences found in T cells affected by various pathological conditions, with TCR sequences associating with their corresponding antigen targets, pathological conditions, and organs [71]. Moreover, ATLAS [91] and TCR3d [92] collect information on TCR structures and the interactions between pMHCs and TCRs.

Most database-provided peptides are derived from pathogens. Consequently, very few such peptides are tumor-specific, resulting in a scarcity of tumor-specific data for studying pMHC recognition. This scarcity leads to significant challenges for the development of related computational methods. Machine learning–based methods are particularly impacted by these challenges, so transfer learning has been widely used to mitigate these challenges [77, 93].

## Overall neoantigen prediction

The five previous sections described the individual components of a bioinformatics pipeline to predict neoantigens. The overall pipeline integrates these individual steps to detect and prioritize candidate neoantigens. Prioritization ranks candidates with greater therapeutic potential, typically those with higher predicted immunogenicity, ahead of the other candidates. Even if the computationally challenging steps are perfectly implemented, building a complete pipeline to detect and prioritize neoantigen candidates accurately remains a significant challenge because we have to combine the outputs generated by these steps into a score representing neoantigen priority. For example, there are many ways to use agretopicity and foreignness, the two aforementioned TCR-unspecific features, and finding such a good way is not trivial.

A typical neoantigen prediction pipeline processes WGS data, WES data, RNA-seq data, and/or peptide FASTA files to predict HLA class I neoantigens from SNVs, InDels, gene fusions, and splicing variants (Table 4). The pipeline typically uses hard filtering at each step to exclude low-priority neoantigen candidates. For example, the pipeline may filter out a candidate with a VAF below 0.05, a predicted pMHC binding affinity above 500 nM, or an estimated neoantigen abundance below 1.0 TPM. Additionally, the pipeline may assign higher priorities to candidates generated by driver and/or clonal mutations, as driver mutations increase the survival and proliferation of cancer cells, and clonal mutations are present in all cancer cells [94]. Commonly used bioinformatics pipelines for overall neoantigen prediction, such as pVACtools [95], VaxRank [96], and OpenVax [97], often integrate the aforementioned databases. Table 6 summarizes the characteristics and applications of these databases in neoantigen prediction.

The state-of-the-art neoantigen prediction pipelines achieved very low performance (Table 5). The Tumor Neoantigen Selection Alliance (TESLA) consortium and Müller *et al.* benchmarked the performance of overall neoantigen prediction [18, 98]. The consortium provided tumor DNA-seq, normal DNA-seq, and normal RNA-seq data from five patients to participating teams. Each team submitted predictions of immunogenic pMHCs for evaluation against immunoassay-generated ground truths. All 25 participating teams performed poorly on the consortium-generated data [18]. After examining the data, the consortium trained a feature selector and a decision tree with five selected features, achieving precision and recall of up to 0.71 and 0.45, respectively, on the training data [18]. In addition to precision and recall, several other metrics can measure neoantigen prediction performance. These include the top-twenty immunogenic fraction (TTIF) [18], fraction ranked [18], area under the precision–recall curve, AUROC, and cohort-level TTIF [98, 99]. Currently, cohort-level TTIF is the most commonly used performance metric [98].

A neoantigen prediction pipeline should not only achieve high performance but also be user-friendly, adhering to principles of data provenance, portability, scalability, and reentrancy [100]. We can ensure these features via the use of bioinformatics workflow managers such as Snakemake [101] or NextFlow [102]. Additionally, pipelines often depend on multiple third-party software packages, making the use of package managers such as bioconda [103] essential for automated, reproducible, and cross-platform installations.

The ground truth of immunogenicities, which are used for prioritizing neoantigens, is based on immunological validation, which remains scarce, especially for cancer-derived peptides [104]. Some key datasets include the following:

1. A study manually curated ~5000 pMHC pairs from both pathogens and cancer patients, with each pMHC experimentally tested to be either immunogenic or nonimmunogenic [44].
2. Four studies reported 435 pMHCs from 15 cancer patients, all of which were experimentally tested for immunogenicity [105–108].
3. The TESLA consortium data included 918 pMHCs from nine patients, 44 of which were experimentally determined to be immunogenic [18]. TESLA also released the raw sequencing data (consisting of bulk tumor WES, normal WES, and tumor RNA-seq) for five patients to the public [18].
4. The harmonized datasets of the National Cancer Institute, TESLA, and Human Integrated Tumor Immunology Discovery Engine feature 178 immunogenic and 421 085 nonimmunogenic neopeptides from 99 patients, where the neopeptides were experimentally tested for immunogenicity [98].

The immunological validation typically uses either pMHC multimer staining or interferon-γ enzyme-linked immunospot (IFN-γ ELISpot) assays. The pMHC multimer staining assay first collects TCRs that are bound to a given pMHC of interest and then detects the TCR-associated T cells by staining. The IFN-γ ELISpot assay measures the expression of IFN-γ by T cells in the presence of a peptide of interest, as this expression indicates the activation of a subset of the T cells.

## Practicality of neoantigen prediction in clinical applications

To enhance the characterization of neoantigens, we can perform additional analyses beyond standard prediction tasks in clinical settings. These analyses can identify driver mutations, compute the tumor mutation burden, infer tumor subclones, conduct phylogenetic analyses of tumor subclones, trace tumor evolution, and predict treatment responses to neoantigen-based therapies. These analyses can be facilitated by resources such as TCGA, a comprehensive database that provides extensive data on cancer-related genetic mutations, enabling insights from a data-driven perspective [27].

Clinicians typically use the top-20 ranked peptides as a mixture for designing neoantigen-based therapy [18]. Unfortunately, for each patient, only less than half of immunogenic peptides are found in the top-20 ranked peptides (Table 5). Therefore, the overall neoantigen prediction as a whole has to be improved. Additionally, a high performance in neoantigen prediction is only part of all the requirements for successful neoantigen-based therapies. For example, the manufacturing process, therapy type (e.g. messenger RNA (i.e. mRNA) vaccine, peptide vaccine, dendritic-cell, and TCR-T therapy), combination with other therapies, and the characteristics of the patient-specific immune system all influence the performance of the neoantigen-based therapy [2, 3, 109, 110]. Therefore, the performance of neoantigen prediction has to be assessed in conjunction with such other factors. Recently, a few clinical trials about neoantigen-based therapies began [110]. We recommend researchers in this field to check the trial results as soon as possible once they are available.

## Current challenges and future perspectives

The computationally challenging steps in neoantigen prediction have varying levels of performance. The state-of-the-art methods have achieved high performance in HLA typing and RNA transcript quantification, medium performance in somatic variant calling and pMHC presentation prediction, and low performance in pMHC recognition prediction (Table 5). Improving pMHC recognition prediction requires a better understanding of the interactions between pMHCs and TCRs, as well as a more comprehensive characterization of a patient’s TCR repertoire [90]. The fulfillment of this need has the potential to significantly improve neoantigen prediction [111]. Additionally, the relationship between neoantigen quality and the result generated by each computationally challenging step remains to be elucidated. After completing the individual steps, we still need to integrate their outputs to predict neoantigens. Therefore, a neoantigen prediction pipeline that utilizes these steps to sensitively detect and accurately prioritize neoantigen candidates is highly desirable [98]. In our recent study, we attempted to employ custom-designed machine learning techniques to improve this prioritization [112].

Neoantigens often exist in only a subset of cancer subclones, but neoantigen-based immunotherapies should target all cancer subclones [2]. Hence, multiregional sequencing (MR-seq) has been applied to estimate the clonal fraction (CF) of each neoantigen, representing the fraction of cancer cells responsive to this neoantigen [113]. However, single-cell sequencing (SCS) is the gold-standard technology for revealing the subclone architecture of the cancer cells of interest [114, 115]. Despite its potential, SCS has not been widely adopted in clinical settings because of its high sequencing cost and requirement for bioinformatics expertise. As the sequencing cost decreases and large language models gain more bioinformatics expertise [116], SCS may replace MR-seq, introducing SCS-related computational challenges to neoantigen prediction.

Currently, neoantigen-based immunotherapies focus on neoantigens generated by stable genomic aberrations (e.g. SNVs and InDels). Emerging evidence suggests that aberrant transcriptomic variants (e.g. RNA splicing variants) and posttranslational modifications could serve as novel sources of neoantigens [2, 117–119] (as reviewed by Cheng *et al*. [120] and Wang *et al*. [121]). These additional sources have the potential to expand the pool of neoantigen candidates for neoantigen-based immunotherapies [122]. However, the immunogenicity and clinical efficacy of these novel candidates remain largely unexplored, requiring further validation [123]. If validated, the computational challenges of identifying these neoantigens—such as detecting splicing variants—will become associated with neoantigen prediction and its applications in immunotherapy, such as anticancer vaccine design.

**Key Points**

- Neoantigen prediction requires the use of many bioinformatics tools, each designed to perform one specific task within the prediction pipeline.
- Every task in neoantigen prediction is computationally challenging, and integrating these task-specific tools into an effective overall pipeline is even more demanding.
- Advances in computational methods for these tasks can drive progress in developing neoantigen-based personalized immunotherapy.
- The increasing availability of data has made the development of advanced computational methods increasingly feasible and necessary for improving neoantigen prediction.

## Data Availability

No data or code was used in this study.
